# Supplementary material for: Remote Implementation of a School-Based Health Promotion and Health Coaching Program in Low-Income Urban and Rural Sites: Program Impact during the COVID-19 Pandemic
Source: Int J Environ Res Public Health. 2023 Jan 6;20(2):1044. doi: 10.3390/ijerph20021044 (PMC9858733; doi:10.3390/ijerph20021044)
Supplement: Supplementary file 1 [file ijerph-20-01044-s001.zip › ijerph-2111913-supplementary.pdf]

Supplementary Materials

Table S1. Comparison of pretest characteristics of participants lost to follow up (N=56) with participants in final study sample (N=100).

	Combined Sample with matching pre and post-test N=100	Unmatched/Incomplete or missing post-tests (lost to follow up) N= 56*
Gender		
• Male	16 (16.0%)	20 (35.7%)
• Female	84 (84.0%)	28 (50%)
Grade		
• 9th	16 (16.0%)	5 (8.9%)
• 10th	14 (14.0%)	12 (21.4%)
• 11th	45 (45.0%)	17(30.4%)
• 12th	25 (25.0%)	15 (26.8%)
Age (mean age in years)	16.05 years	16.16 years
Ethnicity		
• Hispanic or Latino	40 (40%)	26 (46.4%)
Race		
• American Indian or Alaska Native	1 (1%)	3 (5.4%)
• Asian	28 (28.0%)	4 (7.1%)
• Black or African American	3 (3.0%)	3 (5.4%)
• Native Hawaiian or Pacific Islander	1 (1%)	0
• White	37 (37.0%)	26 (46.4%)
• Two or more races	7 (7.0%)	0
• Declined to respond	23 (23.0%)	11 (19.6%)
Other Sample Characteristics		
• Live within 15 minutes to place where shop for food	77 (77%)	33 (58.9%)
• Access to fresh fruits and vegetables most times	93 (93%)	40 (71.4%)
• Migrant work not main source of family income	87 (87%)	36 (64.3%)

*Data missing for participants

Table S2. Pre and Post-test change in PAM® 10 scores by levels, N=92, CA and MO, 2020-21

Initial PAM® level of group	Original Level 1*	Original Level 2*	Original Level 3*	Original Level 4*
Survey Counts	5	18	56	13
Mean PAM® 10 score (**pre-test**)	43.04	50.55	61.94	81.85
Mean PAM® 10 score **(post-test)**	67.40	62.93	73.96	86.11
Mean point change from pre-test	24.36	12.38	12.02	4.26
**% Improved (moved up a level(s))**	**100% (5)**	**66.6% (12)**	**42.8% (24)**	**0% (0)**
% Unchanged	0% (0)	22.2% (4)	55.35% (31)	77% (10)
% Decrease	0% (0)	11.1% (2)	1.8% (1)	23% (3)

* Activation Level Explanations

Level 1: Disengaged and overwhelmed; individuals are passive and lack confidence. Knowledge is low, goal orientation is weak, and adherence is poor. Perspective: "My doctor is in charge of my health."

Level 2: Becoming aware, but still struggling; individuals have some knowledge, but large gaps remain. They believe health is largely out of their control but can set simple goals. Perspective: "I could be doing more."

Level 3: Taking action; individuals have the key facts and are building self-management skills. They strive for practice behaviors and are goal oriented. Perspective: "I'm part of my health care team."

Level 4: Maintaining behavior and pushing further; individuals have adopted new behaviors but may struggle in times of stress or change. Maintaining a healthy lifestyle is a key focus. Perspective: "I'm my own health advocate."

Source: Patient Activation Measure®(PAM®), available on https://www.insigniahealth.com/products/pam
